# The Electronic Faces Thermometer Scale (eFTS)—Construct Validity for Pain Assessment in Pediatric Postoperative Care in Sweden

**DOI:** 10.1111/pan.70056

**Published:** 2025-09-22

**Authors:** Angelica Höök, Mia Hylén, Maria Björk, Stefan Nilsson, Jinbing Bai, Henrik Berlin, Helena Hansson, Gudrún Kristjánsdóttir, Rikard Roxner, Pernilla Stenström, Charlotte Castor

**Affiliations:** ^1^ Institute of Health and Care Sciences, Sahlgrenska Academy University of Gothenburg Gothenburg Sweden; ^2^ University of Gothenburg Centre for Person‐Centred Care (GPCC), Sahlgrenska Academy University of Gothenburg Gothenburg Sweden; ^3^ Department of Intensive and Perioperative Care Skåne University Hospital Malmö Sweden; ^4^ Department of Health Sciences, Faculty of Medicine Lund University Lund Sweden; ^5^ The CHILD Research Group, Department of Nursing, School of Health and Welfare Jönköping University Jönköping Sweden; ^6^ Queen Silvia Children's Hospital Sahlgrenska University Hospital Gothenburg Sweden; ^7^ Nell Hodgson Woodruff School of Nursing Emory University Atlanta Georgia USA; ^8^ Department of Pediatric Dentistry, Faculty of Odontology Malmö University Malmö Sweden; ^9^ Department of Paediatrics and Adolescent Medicine Copenhagen University Hospital Rigshospitalet Copenhagen Denmark; ^10^ Department of Clinical Medicine University of Copenhagen Copenhagen Denmark; ^11^ Faculty of Nursing and Midwifery, School of Health Sciences University of Iceland Reykjavik Iceland; ^12^ Hringur Children's Hospital Landspitali University Hospital Reykjavik Iceland; ^13^ Department of Pediatric Surgery, Skåne University Hospital Lund University Lund Sweden; ^14^ Department of Paediatrics, Faculty of Medicine Lund University Lund Sweden; ^15^ The Institute for Palliative Care Lund University, Region Skåne Lund Sweden

**Keywords:** e‐health, pain assessment, pediatric, validation postoperative

## Abstract

**Background:**

Pain in children is undertreated. An assessment scale co‐designed with children, parents, and health care professionals could lead to more effective pain assessments and treatment strategies aimed at reducing pain and pain‐related symptoms. There are analogue scales validated for self‐report of pain in children, but today, children regularly use digital technology, which healthcare should align with. The newly developed electronic Faces Thermometer Scale is a digital assessment scale that needs further validation before it may be recommended for self‐reporting pain intensity.

**Aims:**

The study aimed to determine the convergent and discriminant validity of a new digital pain assessment scale in a pediatric postoperative setting.

**Methods:**

The study was performed at a pediatric surgery department in southern Sweden. A total of 88 children were included, generating 716 assessments. Convergent validity was established by comparing the well‐validated Colored Analogue Scale and Faces Pain Scale Revised with the electronic Faces Thermometer Scale. Pain assessments were conducted at three different time points: one before surgery, one once the participant became alert and aware, and one 30–45 min after the second time point. A *p*‐value of 0.05 was considered statistically significant. Discriminant validity was established by comparing a potential non‐painful situation with a painful situation using the electronic Faces Thermometer Scale.

**Results:**

The agreement between the scales at different time points, as well as across different ages and gender, showed a statistically significant correlation: Kendall's Tau *B* correlation coefficient varied between 0.61 and 0.79 at different time points. The electronic Faces Thermometer Scale was able to discriminate pain across different age groups and genders. There was a statistically significant difference between pre‐ and postoperative assessments, and the Clopper–Pearson proportion ranged from 0.70 to 0.90.

**Conclusions:**

The electronic Faces Thermometer Scale provides a valid digital scale for self‐report of pain within pediatric postoperative care.

## 1 Introduction

Making the pain of children (aged 0–17) visible by assessing with valid instruments is an essential step in providing pain relief [1]. Pain is mainly subjective and should, therefore, primarily be self‐reported [2]. Further, effective and safe pain management includes systematic pain assessments, which may also decrease the risk of chronic pain [3].

Safeguarding children's rights and encouraging them to express themselves on matters that directly affect them will require care tailored to their preferences [4]. A valid self‐report assessment scale has been shown to support children in effectively communicating their pain and evaluating the outcomes of a given pain treatment [1]. In a postoperative setting, pain assessment is recommended for identifying children who perceive themselves as needing pain medication and to evaluate their satisfaction with the given pain treatment. Despite the existence of validated assessment scales for the adequate assessment and treatment of pain, they are not sufficiently utilized by healthcare professionals [5].

Although validated analogue scales for self‐reported pain in children exist, exposure to digital technology and tools means children now grow up as digital natives, with technology as a part of their daily lives. Thus, the use of digital technology and tools for pain management may alter standard care, as children tend to prefer digital media [6, 7].

Pain assessment scales must accommodate the dynamic and individually variable nature of pain experiences. Construct validity, comprising convergent and discriminant validity, assesses how effectively an instrument measures its intended theoretical construct. Moreover, validated assessment scales are important to ensure that patient‐reported outcome measures are effective, reliable, and relevant for capturing patient experiences in healthcare settings [8].

The electronic Faces Thermometer Scale (eFTS) is an assessment scale developed to assess different symptoms (e.g., intensity and how the symptom affects you), including pain, and is inspired by previously validated scales. Developing an assessment scale designed in collaboration with children may be an effective strategy for empowering them as active participants in their care. The eFTS is part of the mobile application, Pictorial Support in Person‐Centered Care for children (PicPecc), developed through co‐design with children, parents, and health care professionals [9].

The eFTS has shown potential for use in pain assessments, and children reported that it facilitated their communication about pain [10]. Evidence‐based recommendations for self‐reported postoperative pain in children aged 8 years and older include (among others) the Faces Pain Scale‐Revised (FPS‐R) and the Colored Analogue Scale (CAS) [11]. These scales are commonly used within Swedish pediatric healthcare. Further, the FPS‐R is recommended by the International Association of the Study of Pain (IASP) for pain assessments in children [12]. To ensure quality, the eFTS needs to be tested for agreement with validated scales previously used in pediatric postoperative care, as well as for its ability to discriminate pain [13].

### 1.1 Aim

This study aimed to determine the convergent and discriminant validity of a new digital pain assessment scale in a pediatric postoperative setting.

Research question:

- Is there agreement between the eFTS and validated analogue scales, CAS and Face Pain Scale‐Revised (FPS‐R), in the whole sample and age and gender subgroups, respectively?
- Does the eFTS have the ability to discriminate pain at different time points, in the whole sample and age and gender subgroups, respectively?

## 2 Methods

### 2.1 Design

This observational study, consisting of repeated measurements, compared eFTS with CAS and FPS‐R. It is part of a cross‐sectional, multi‐site project evaluating the psychometric properties of eFTS from different perspectives and in various settings and deviates from the study protocol published in 2023 [13].

### 2.2 Setting

The study was conducted between April 2021 and May 2023 at a designated national department of surgery for children at a university hospital in southern Sweden. Participants were recruited in the surgical outpatient unit, which cares for up to 45 children aged 0–17 years every week. The department covers pediatric surgery, orthopedics, ophthalmology, and ear, nose, and throat surgery. Orthopedic procedures are the most frequent.

### 2.3 Participants

A convenience sample of children aged 8–17 was applied. The inclusion criteria were children scheduled for a surgical procedure with planned postsurgical recovery and able to understand and read Swedish. Exclusion criteria were children or their parents who did not understand the instructions, as well as children who could not self‐report their pain. Children with clinical instability (e.g., end‐of‐life care or illness necessitating admission to intensive care) were excluded. The attending nurse identified the participating children upon their arrival in the outpatient unit.

### 2.4 Sample Size

According to the sample size calculations described by Castor et al. [13], an estimated volume of approximately 30 participants was planned for each group. To compare agreements in subgroups (age and gender), a minimum sample size of 60 children was required. To compensate for a heterogeneous sample regarding types of surgery (i.e., length and pain intensity) and missing data, 85 children were estimated to be a sufficient sample size. This was supported by Pagé et al. [14], who aimed to examine convergent and discriminant validity in a postoperative setting that included 83 children aged between 8 and 18.

### 2.5 Instruments

#### 2.5.1 Digital Instruments

**FIGURE 1:**
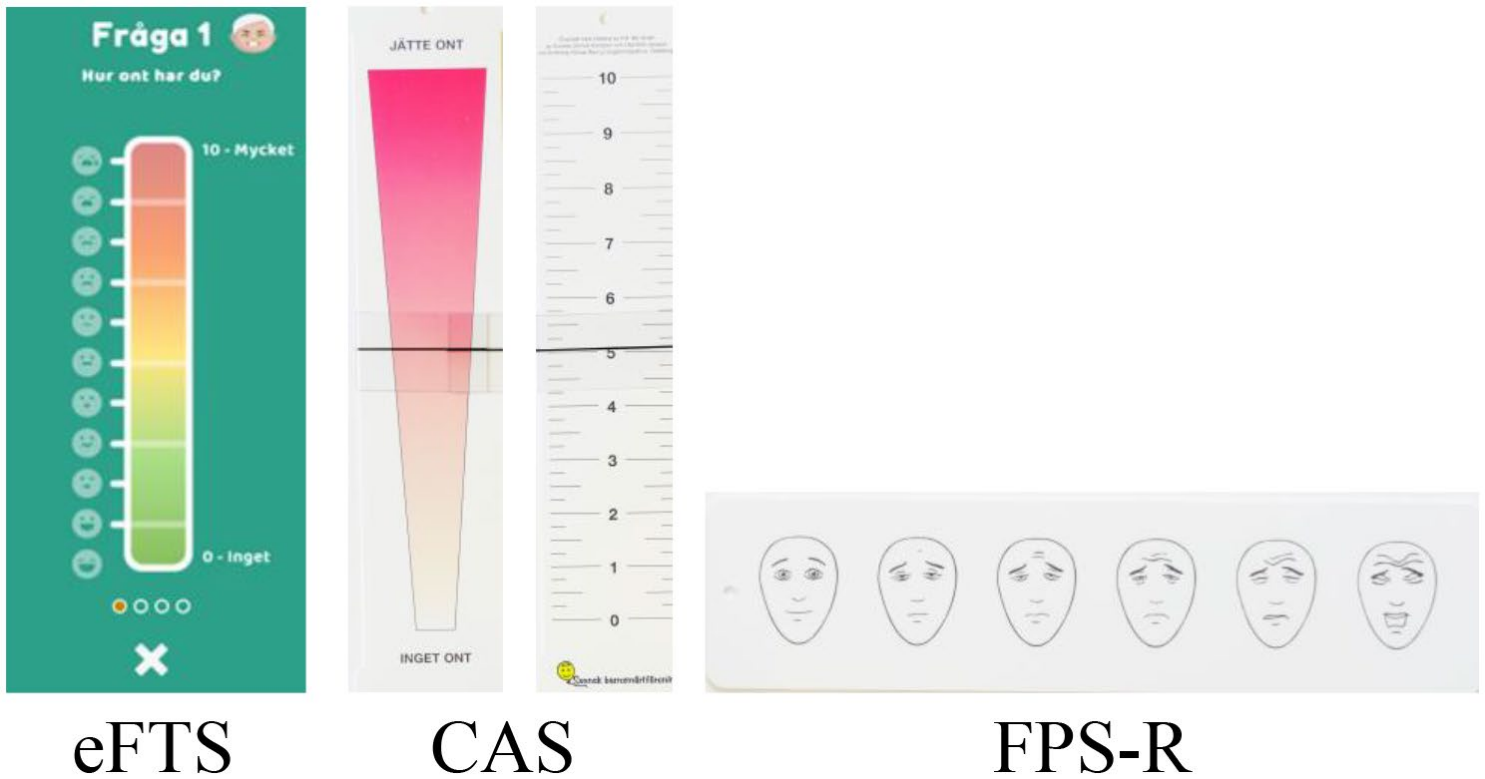
The electronic Faces Thermometer Scale (eFTS), Colored Analogue Scale (CAS), and Faces Pain Scale‐Revised (FPS‐R).

The eFTS is a digital vertical assessment scale using (a) colors—green to red, (b) lines representing 0 to 10, and (c) face‐emojis—happy to sad, to visualize pain intensity. End points are explained using the words “nothing” and “very much” (Figure 1).

Participating children were introduced to eFTS on a tablet computer with a 21 × 16 cm screen, and the height of the scale on the screen was 12 cm. The clinician began by reading the following instruction: “This scale shows how much pain one can have. Down here means no pain at all. The scale indicates an increasing level of pain, which is quite painful here. Now, show me on the scale how much pain you feel right now.” After this, the participating children assessed their pain intensity by pointing to the eFTS with their fingers to select the level that best represented their pain intensity.

#### 2.5.2 Analogue Instruments

The CAS is a vertical plastic strip with a wedge‐shaped color‐gradated figure on one side and a numerical scale on the other. The color‐gradated figure ranges from white and narrow (no pain) to red and wide (the most pain) and is scored from 0 to10 in increments of 0.25, with a movable slide [15]. Children were shown the side of the instrument, with the wedge‐shaped figure and the slider positioned in the middle. The clinician then instructed the child: “Move the slider to the place that shows how much pain you have. This means you have no pain (slider is at the bottom). This end means you have the worst pain (slider moved to the top).” The slider was moved back to the middle of the scale before the children used it, according to the protocol described by McGrath et al. [15].

The FPS‐R consists of six faces on a horizontal plastic strip. The faces are gender‐ and race‐neutral, depicting expressions of pain, with “no pain” (neutral, furthest left) to “very much pain” (furthest right) representing an increasing degree of pain moving from left to right, accompanied by a corresponding numerical score from 0 to 10 in increments of 2. Each child was shown the faces on the strip and instructed to select the face that symbolized the intensity of pain [16].

All the scales were used as intended, that is, the eFTS as an eleven‐step scale (0–10, in units of 1), the CAS as an eleven‐step scale (0–10, in units of 0.25), and the FPS‐R as a six‐step scale (0–10, in units of 2).

### 2.6 Procedure and Data Collection

Demographic data, such as age and gender, were collected from participating children or their parents using a paper questionnaire. An additional questionnaire included information on surgical interventions, type of anesthesia, and pain management before, during, and after surgery, which nurses completed. Participating children were assigned to one of six different groups. Each group had a predetermined order for assessing the scales, which was randomly defined to avoid assessment bias.

**FIGURE 2:**
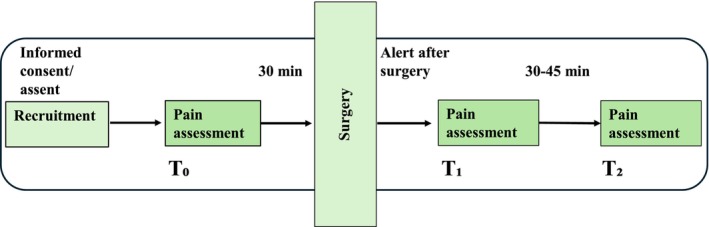
Flowchart for data collection.

Pain assessments were conducted at three different time points. The first assessment (*t*_0_) was conducted before surgery, the second (*t*_1_) when the participant was alert and aware, and the third (*t*_2_) was performed 30–45 min after the second time point (Figure 2).

### 2.7 Statistical Methods

Descriptive statistics, including mean (standard deviation [SD]) and frequency [%], were calculated for demographic variables. To enable comparison between the three scales, an equating procedure was performed [17] aligning the lowest 25% from FPS‐R assessments with the lowest 25% scores from eFTS and CAS, respectively. The same procedures were used for scores up to 50% and up to 75%.

Children were grouped by age (8–12 years and 13–17 years) [18], as well as gender. Convergent validity between the eFTS, CAS, and FPS‐R was assessed using Kendall's Tau *B*, suitable for small subgroups. Analyses were made to determine whether agreements between the scales would depend on these factors. Threshold values for strong and very strong correlation were set to 0.5 and 0.7 respectively [19].

For discriminant validity, analyses were performed to determine whether pain intensity changed over time [20]. The Clopper–Pearson exact method was used to calculate confidence intervals around the proportion who reported an increased pain intensity level between *t*_0_ and *t*_1_. This method is suitable for constructing confidence limits for data where observations have two possible outcomes (success for failure) in small sample sizes. To support discriminant validity, a minimum difference criterion of 50% (0.5) was applied to the observed proportion of participants with increased pain intensity in order to determine that the success rate was not likely due to chance.

Analyses were completed using the Statistical Package for Social Sciences (IBM SPSS Statistics 29.0.0).

### 2.8 Ethical Considerations

The Swedish Ethical Review Board approved the study (Dnr: 2020‐05119). Potential participants and their legal guardians received age‐appropriate oral and written information about the study prior to signing a standard informed consent form. For participants under 15 years of age, the legal guardian signed the form while the participant provided their assent. Participants aged 15–17 signed the informed consent form themselves.

## 3 Results

Eighty‐eight (*n* = 88) children were included. Two children did not complete any of the assessments leading to exclusion from the study. Table 1 presents the descriptive statistics of participants.

**TABLE 1:** Descriptive statistics of the participants.

Category	*N* (%)	Mean age	SD
All participants	86 (100)	12.78	2.14
Sex			
Female	40 (46.5)	12.56	2.13
Male	46 (53.5)	13.13	2.03
Age group			
8–12‐year‐old	34 (39.5)	10.67	1.21
13–17‐year‐old	52 (60.5)	14.23	1.12

### 3.1 Characteristics of Participants

Participants received general anesthesia, and their surgeries lasted 10–120 min (mean = 48 min, SD = 26.5). The operating procedures varied from minor procedures, such as teeth extractions or orchidofunicolysis, to more extensive orthopedic or tumor surgery.

Of the participating children, 53 (62%) received premedication with a long‐acting opioid, and 6 (7%) also received benzodiazepine. All children received perioperative analgesia containing paracetamol and parecoxib, and 55 (64%) received local anesthesia (ropivacaine or bupivacaine). Postoperative pain treatment consisting of intravenous opioid and/or clonidine was given to 34 (40%) of the participating children. Premedication and perioperative analgesia were administered according to local protocols specific to the type of surgery performed.

### 3.2 Pain Assessments

There were 226 assessments on eFTS with a median value of 3.0 (range: 0–9; IQR = 4.0). CAS had 247 assessments with a median value of 2.0 (range: 0–9; IQR = 4.0) and FPS‐R had 243 assessments with a median value of 2.0 (range: 0–8; IQR = 4.0).

### 3.3 Convergent Validity

All assessments showed a positive correlation between the eFTS and both the comparative analogue scales (CAS and FPS‐R). The agreement was consistent when divided into age‐ and gender‐specific subgroups.

**FIGURE 3:**
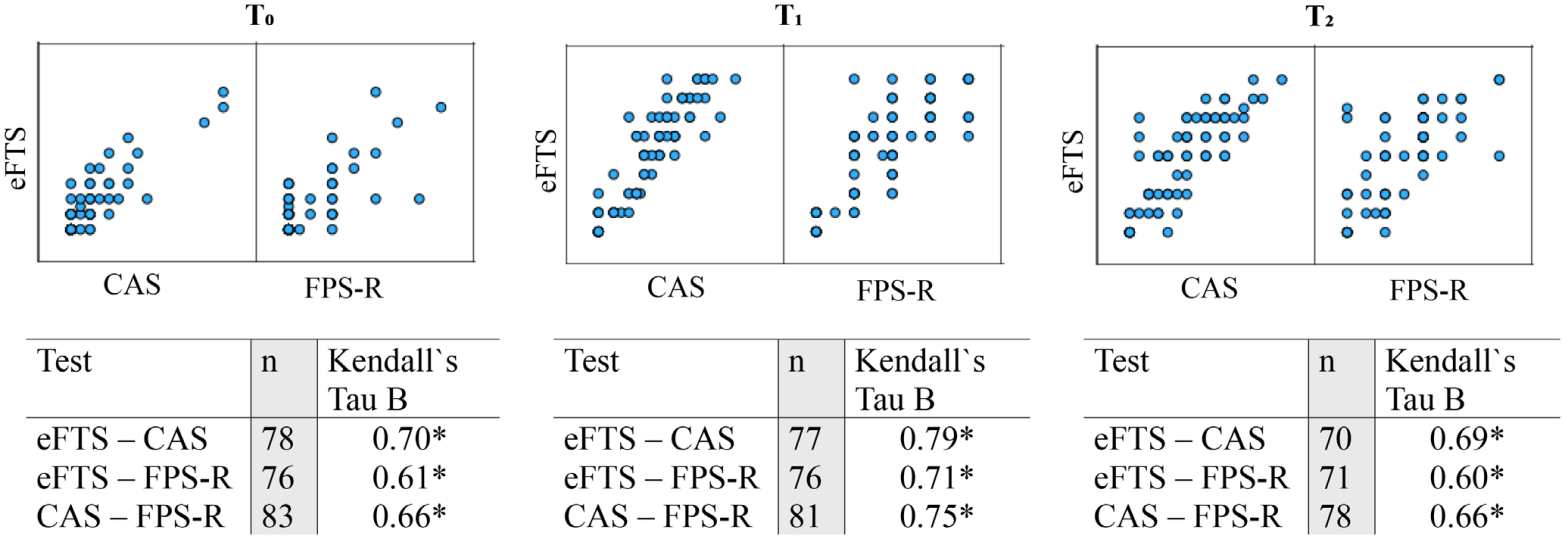
Scatter plot and correlation coefficient for different time points. *p < 0.001.

Kendall's Tau *B* correlations between eFTS, CAS, and FPS‐R at the different timepoints displayed a stronger correlation at *T*_1_ than at *T*_0_ and *T*_2_. All correlations showed statistical significance at the *p* < 0.001 level (Figure 3).

When divided into age groups, the correlation was stronger between eFTS and CAS at *T*_0_ and *T*_1_ in both age groups, and in the older age group at *T*_2_, than between eFTS and FPS‐R. All groups had a statistically significant correlation at different time points (Table 2).

**TABLE 2:** Correlation coefficients in subgroups of age and gender.

Kendall's Tau *B*	eFTS					
	*T*_0_		*T*_1_		*T*_2_	
**Age**	**(*n*) 8–12**	**(*n*) 13–17**	**(*n*) 8–12**	**(*n*) 13–17**	**(*n*) 8–12**	**(*n*) 13–17**
CAS	(31) 0.67*	(47) 0.72*	(30) 0.77*	(47) 0.82*	(26) 0.49*	(44) 0.77*
FPS‐R	(30) 0.53*	(46) 0.66*	(29) 0.72*	(47) 0.65*	(26) 0.65*	(45) 0.66*
**Gender**	**(*n*) Male**	**(*n*) Female**	**(*n*) Male**	**(*n*) Female**	**(*n*) Male**	**(*n*) Female**
CAS	(41) 0.57*	(37) 0.81*	(41) 0.87*	(36) 0.68*	(39) 0.76*	(31) 0.58*
FPS‐R	(40) 0.46*	(36) 0.76*	(41) 0.71*	(35) 0.59*	(39) 0.76*	(32) 0.47*

* *p* < 0.001.

There was also a statistically significant correlation when divided by gender, with a stronger correlation for females at *T*_0_ than males. In comparison, the correlation for males at *T*_1_ and *T*_2_ was stronger than for females.

### 3.4 Discriminant Validity

Of the 86 participants, 73 assessed their pain using eFTS at both *T*_0_ and *T*_1_. There was a statistically significant difference between timepoints *T*_0_ and *T*_1_, with higher assessments at *T*_1_. The proportion of successes exceeded 50%, which was the difference criterion. When divided into gender and age groups, more girls than boys assessed their pain intensity to be higher at *T*_1_ than at *T*_0_. Similarly, the participating children in the older age group assessed their pain intensity to be higher at *T*_1_ than at *T*_0_ than those in the younger age group (Table 3).

**TABLE 3:** Confidence intervals for proportions using the Clopper–Pearson method.

	*n*	Clopper–Pearson proportion	95% Confidence interval		*p*
			Lower	Upper	
Total	73	0.78	0.67	0.87	< 0.001
Gender					
Male	38	0.70	0.51	0.82	0.03
Female	35	0.90	0.73	0.96	< 0.001
Age group					
8–12	28	0.75	0.55	0.89	0.013
13–17	45	0.80	0.65	0.91	< 0.001

## 4 Discussion

This paper reports on the convergent and discriminant validity of the eFTS for pain assessments in children aged 8–17 years old. The study demonstrated that eFTS is equivalent to the established analogue assessment scales, CAS and FPS‐R, in terms of self‐reporting pain in children. A statistically significant correlation existed between eFTS and CAS, as well as between eFTS and FPS‐R, at different time points. When the data were split into different subgroups of age and gender, these correlations were still strong. Previous studies also support the agreement between electronic and analogue methods for capturing pain‐related data. These studies support the use of apps and highlight the interchangeability between electronic and analogue methods for self‐reporting pain. Additionally, they emphasize the practicality and effectiveness of electronic methods for collecting pain‐related data [21, 22].

In terms of pain assessment, the scales varied in range, with children rating their pain intensity higher (0–9) when using eFTS and CAS compared to FPS‐R (0–8). One possible explanation is that CAS does not use numerical grading at all, and FPS‐R only employs the numbers 2, 4, 6, and 8. In contrast, eFTS offers more options, allowing the child to rate their pain on a scale of 0–10 in visual increments. This warrants further investigation in future studies. The findings also indicated a slight difference in the median value when using eFTS compared to CAS and FPS‐R. This discrepancy may be related to the transition between analogue and digital assessment scales, suggesting that pain assessment scales should not be used interchangeably in a child's care.

The agreement between eFTS and FPS‐R was weaker than the agreement between eFTS and CAS.

With the eFTS, alternative ways of visualizing pain intensity (color, face‐emojis, and numbers) are used, whereas the FPS‐R only uses faces to visualize pain intensity. In eFTS, a smiling face symbolizes no pain, whereas in FPS‐R, a neutral face symbolizes no pain. There are different views on using smiling anchors in pain intensity scales [16, 23]. This study employed scales with both a smiling anchor and a neutral anchor, which may have confused the participating children and contributed to the weaker agreement between eFTS and FPS‐R.

Self‐reporting of pain is the gold standard, and visualization could support what is needed to make pain visible. However, the use of a validated scale for pain does not necessarily make a difference in hospitalized children with pain. Further, visualized pain also needs to be acted upon by healthcare professionals [24]. Birnie et al. [25] identified that children and parents believed an app for self‐management (including assessments) of postoperative pain in a home setting could be beneficial.

A digital assessment scale such as the eFTS may support pain communication across settings and enable the early identification of inadequate pain management. The use of a validated scale outside the hospital may facilitate home‐based postoperative care through structured and remote pain monitoring.

Our findings support the potential application of eFTS as a scale for self‐reporting postoperative pain, and further studies are required to evaluate its effectiveness in various inpatient and outpatient settings, as well as in populations with cognitive variations.

### 4.1 Strengths and Limitations

This study was conducted at a single institution in Sweden, which limits the immediate generalizability of the results. However, the new knowledge adds to the collective validation and generalizability of the eFTS in different contexts.

One strength of this study was the design, which allowed for the inclusion of various settings and medical conditions in the sample. Despite the heterogeneity of the sample, one way to structure a study like this is to assign participants to different groups based on the order in which the assessment scales are presented, as has been done here.

The use of analogue scales for the validation of a digital scale may be a limitation: our intention was to employ the analogue scales considered the gold standard within pediatric healthcare.

The use of Kendall's Tau *B* resulted in a lower correlation coefficient than studies using different analyses, such as the Pearson correlation coefficient, for validating pain assessment scales [26]. Using Kendall's Tau *B* may complicate comparisons between validation studies regarding correlation coefficients, as cut‐off values for strong and very strong correlation differ between methods [19]. Despite these issues, the limited sample sizes within subgroups made Kendall's Tau *B* correlation coefficient a suitable test.

The Clopper–Pearson method was used for determining discriminant validity by calculating the proportion of participants who assessed their pain as higher at *T*_1_ than at *T*_0_. This approach might make it difficult to compare our results with studies correlating scales measuring different symptoms (e.g., pain and nausea). This method was still deemed appropriate for the aim of this study.

Finally, this study constitutes only one part of a series of published [10] and ongoing studies evaluating the validity of the eFTS and deviates from the study protocol [13]. Even though significant adjustments have been made in relation to the proposed study [13] we consider the existence of a structured protocol taking different aspects of validity and reliability into account as a strength.

## 5 Conclusions

The eFTS demonstrates convergent and discriminant validity for pre‐ and postoperative pain assessments in children and can be considered a valid and accessible scale for self‐reporting pain intensity within pediatric postoperative care. Together with previous findings on content validity, we suggest that the eFTS enhances children's ability to assess their pain intensity independently. This may provide more accurate, timely, and equitable assessments, facilitating pain management.

## Author Contributions

M.B., S.N., J.B., H.H., G.K., P.S., and C.C. initiated and developed the study design. C.C. was responsible for the ethics application and organized data collection. A.H., M.H., H.B., and R.R. contributed to parts of the design. A.H., with the support of M.H., was responsible for data management, including statistical analysis and data interpretation. Additionally, A.H. and M.H. collaboratively drafted the manuscript, which was subsequently revised, developed, and finalized with contributions and agreement from all authors. All authors have read and agreed to the published version of the manuscript.

## Conflicts of Interest

The authors declare no conflicts of interest.

## Data Availability

The datasets presented in this article are not readily available because the General Data Protection Regulation (GDPR) applies to the data collected in this study, that is, any information that refers to an identified or identifiable natural person. The GDPR applies in principle to every kind of operation and activity, regardless of who processes this personal data. Thus, it applies to companies, associations, organizations, authorities, and private individuals. Requests to access the datasets should be directed to angelica.hook@gu.se.
